# Displacement-related factors influencing marital practices and associated intimate partner violence risk among Somali refugees in Dollo Ado, Ethiopia: a qualitative study

**DOI:** 10.1186/s13031-020-00267-z

**Published:** 2020-04-07

**Authors:** Vandana Sharma, Adaugo Amobi, Samuel Tewolde, Negussie Deyessa, Jennifer Scott

**Affiliations:** 1 Harvard T.H. Chan School of Public Health, 677 Huntington Avenue, Boston, MA 02115 USA; 2 Harvard Medical School, Boston, USA; 3 Massachusetts General Hospital, Boston, USA; 4 Women and Health Alliance International, Addis Ababa, Ethiopia and Paris, France; 5 Addis Ababa University School of Public Health, Addis Ababa, Ethiopia; 6 Beth Israel Deaconess Medical Center, Boston, USA

**Keywords:** Somali refugees, Displacement, Early marriage, Forced marriage, Polygamy, Intimate partner violence, Ethiopia, Qualitative research

## Abstract

**Background:**

Child and forced marriage have negative health consequences including increased risk of intimate partner violence (IPV) for women and girls. War and humanitarian crises may impact decision-making around marriage and risks of IPV for displaced populations. A qualitative study was conducted among Somali refugees in Dollo Ado, Ethiopia to understand the interplay of factors that contribute to IPV and to inform an intervention. This secondary analysis aims to explore the influence of displacement on marital practices and associated IPV risk.

**Methods:**

Interviews and focus group discussions were conducted in 2016 in Dollo Ado, Ethiopia, among Somali women and men living in Bokolmayo refugee camp, host community members, non-governmental staff and service providers, stakeholders, and community and religious leaders (*N* = 110). Data were transcribed, translated to English, and coded and analyzed thematically using Dedoose software and a codebook developed a priori.

**Results:**

Findings reveal numerous displacement-related factors that led to perceived shifts in marital practices among refugees, including reductions in child and forced marriages. NGO awareness-raising programs and Ethiopian laws prohibiting child marriage as well as increased access to education for girls were reported to have contributed to these changes, despite continued economic hardship and high perceived risk of non-partner sexual violence within the camp and host community. Polygamy was also perceived to have decreased, primarily due to worsening economic conditions. Forced marriage, polygamy and dowry were reported to contribute to physical IPV, and sexual IPV was reported as common in all types of marital unions. However, there was no evidence that changes in these marital practices contributed to any perceived declines in IPV within this context.

**Conclusion:**

Safe access to education for girls should be prioritized in humanitarian settings. Interventions to address child and forced marriage should address gender and social norms. Intimate partner violence prevention programming should include specialized content taking into account marital practices including child and forced marriage and polygamy. Laws recognizing sexual IPV within marital relationships are needed to reduce sexual IPV.

## Background

Child marriage, defined as a formal or informal union before 18 years of age, is considered a human rights violation and an important international public health issue [1, 2]. Globally, 34% of women aged 20–24 were married before their 18th birthdays, and each day an additional 39,000 girls under 18 are married [3]. There is heterogeneity in the rate of child marriage by country, with the highest levels occurring in sub-Saharan African and South Asian countries. Somalia has one of the highest rates of child marriage, with 45% of women aged 20–24 married before the age of 18, and 8% married before 15 years of age [4]. Child marriage is usually included within the scope of forced marriage, defined as marriages where one or both spouses, regardless of age, did not give full and free consent [5, 6]. Forced marriage is distinct from arranged marriage where family members introduce spouses but both parties provide consent to the union. However, in practice it can be difficult to differentiate between the two and discern at which point emotional or family pressure forces a marriage [7]. The prevalence of forced marriage is difficult to estimate as the practice is under-reported [8].

Child and forced marriages have sexual and reproductive health consequences for women and girls including elevated HIV risk, early and frequent pregnancies, and increased pregnancy complications resulting in disability such as obstetric fistula as well as maternal death [8–14]. Underlying these health outcomes are social norms to bear children at a young age, limited decision-making power within their relationships, and limited access to health services [9, 15]. There is evidence of adverse mental health outcomes among child brides, including depression and anxiety [16, 17]. Research also demonstrates that child marriage places girls and women at risk for intimate partner violence (IPV) [18–21], which in turn increases negative physical and mental health outcomes [22–24]. The higher prevalence of IPV among child brides is likely related to power disparities from spousal age gaps, lower educational attainment, lack of female autonomy and reduced economic opportunities [19, 20]. Furthermore, men who marry young girls may be more likely to hold traditional masculine ideologies that support violence, and therefore may be more likely to perpetrate violence [25].

War and humanitarian crises affect decisions around marriage [26–29]. In humanitarian contexts, child marriage has been reported as a coping and survival strategy for families. For example, quantitative data suggest increases in the incidence of child marriage among Syrian refugees in Lebanon [30], while other research in the same context highlights marriage at an early age as a means to safeguard family reputation and honor and protect girls against sexual violence and the possibility of pregnancy outside of marriage [31, 32]. Among Syrian refugees in Turkey, displacement was linked to changes in the social trajectories of girls, including entry into the workforce, earlier marriage due to financial strain and lack of educational opportunities [33]. In Uganda, conflict resulted in increased barriers to education, separation of families, breakdown of communication between parents and youth and increased poverty, which in turn contributed to changes in relationships and marital practices including earlier relationships and informal marriage among displaced populations [28]. Child marriage may not always occur solely as a result of parental decisions, and some literature calls for more nuanced understanding of adolescents’ roles in marital decisions. For example, in a Palestinian refugee camp in Lebanon, adolescent girls described being actively involved in the decision-making process around their engagements and underlined isolation and post-conflict loss of friends as being the key motivational factors for early marriage [34]. A study conducted in Somalia reported that increased agency of adolescents, along with greater access to technology and poor economic conditions, contributed to increased child marriage that was initiated by the adolescents themselves [35]. In Somalia, armed groups have also imposed child marriage, and there are reports of abduction of girls and forced marriages [36].

Interestingly, research suggests that conflict is not always associated with an increase in the rate of child marriage [37]. For example, age at marriage in some countries that have experienced civil war such as Algeria has remained relatively high [38]. The effect of conflict on child marriage depends on context and varies based on a number of economic, psychosocial and conflict-related factors [37]. There is some evidence that minimum marriage age laws may be associated with a lower prevalence of child marriage [39], but it is not clear how these laws may influence displaced populations.

Refugees and internally displaced populations (IDPs) are also at increased risk for gender-based violence (GBV) including IPV [40, 41]. There is some evidence that changes in marital practices due to displacement may increase risk of IPV [29, 42]. For example, a study among displaced populations in Uganda found that young men were unable to pay dowry and marriages reportedly became less formalized due to economic hardship and disruption of families [29]. The lack of formalization of marriages meant that families were less involved and supportive and this was noted to contribute to isolation of couples and increased risk of emotional and physical IPV among child brides [29]. In addition to child marriage and dowry, other types of marital practices that have been associated with IPV including polygamy [43] may also be impacted by humanitarian crises but there are few studies examining these areas. There have been increasing efforts to develop and evaluate IPV prevention strategies for humanitarian contexts but few interventions have been tailored specifically to address factors related to child and forced marriage or other marital practices. One study in Côte d’Ivoire found differential effects of an IPV prevention intervention which led to improved outcomes in women (non-child brides), but not among women married as child brides suggesting that this population may require specific types of IPV interventions [44].

Overall, there is a need for more evidence on the impact of conflict and displacement on marital practices, including decisions on the age of marriage and how these practices influence IPV risk for women and girls in humanitarian crises. A qualitative study was conducted among Somali refugees in Dollo Ado, Ethiopia to understand the interplay of factors that contribute to IPV and to inform the adaption of the Unite for a Better Life program, an in-person group-based intervention to prevent and reduce IPV [45]. This paper presents a secondary analysis of the data which aims to explore the influence of displacement on marital practices and associated IPV risk.

## Methods

A qualitative study was conducted in October 2016 in Bokolmayo refugee camp in Ethiopia in partnership with Women and Health Alliance (WAHA) International in Ethiopia, Addis Ababa University School of Pubic Health and Beth Israel Deaconess Medical Center (BIDMC). Qualitative data were collected using in-depth interviews (IDIs), focus group discussions (FGDs) as well as group-based participatory learning activities (PLAs) (See Table 1). The PLAs included free listing exercises on GBV, vignettes about GBV as well as community mapping to identify locations where GBV occurs within the camp. The main results of the qualitative study are being reported elsewhere. Focus group discussions and PLAs were included to capture group norms, and similarities and differences in participants’ experiences and opinions. However, some individuals may be reluctant to discuss private or sensitive topics in a group setting, and IDIs were included to capture dissenting perspectives. For the purposes of this paper, we present data from the IDIs and FGDs to analyze changes in marital practices. The PLAs were excluded from this analysis given that they did not include questions related to marital practices.

**Table 1:** Summary of data collection methods for overall study

Methodology	# of Interviews / discussions	# of Participants
In-depth Interview	30	30
Focus Group Discussion	10	80
Participatory Learning Activity* (Free Listing and Vignettes)	10	81
Participatory Learning Activity* (Community Mapping)	3	24
**TOTAL**	**53**	**215**

*data were excluded from this analysis

### Study setting

Bokolmayo is one of five refugee camps near Dollo Ado, Ethiopia, a small town bordering Somalia. The camp opened in 2010 to accommodate refugees from Somalia after years of conflict, unstable governance, and drought led to widespread population displacement. At the time of the study, the United Nations High Commissioner for Refugees (UNHCR) estimated that over 200,000 Somali refugees were registered in the five camps of Dollo Ado, Ethiopia, with 42,385 in Bokolmayo [46].

### Study design and sampling

The sample population comprised four groups: 1) refugee community members, 2) United Nations (UN), non-governmental organization (NGO) and community-based organization (CBO) staff and/or other service providers working in the camp, 3) community leaders and/or elders living in the refugee camp, and 4) host community members. Participants were recruited using purposive sampling and in consultation with UNHCR and the Administration for Refugee and Returnee Affairs (ARRA), and a study community advisory board. The inclusion criteria for refugee community members included: females and males aged 15 and above who identified as refugees from Somalia and who had resided in Bokolmayo camp for at least 6 months. As child marriage is commonly practiced in Somalia, it was important to include respondents less than 18 years of age to understand their perspectives. The inclusion criteria for organizational and/or service providers included: adult (aged 18 and older) female and male staff and service providers from community organizations operating in Dollo Ado, who had worked for at least 1 year for an agency or organization that provides services to refugee populations. The inclusion criteria for community leaders and elders included: adult (aged 18 and older) female and male community leaders and/or religious leaders in Dollo Ado, who had been in Bokolmayo camp for at least 6 months.

### Data collection

Semi-structured questions for each data collection method were developed by study authors to explore the forms of GBV that occur in the camp, as well as the social, religious and cultural factors that contribute to GBV or protect against it, and how displacement may have influenced these factors. Questions assessed cultural practices before displacement, circumstances that threaten the safety of women and girls in the camp, relationships and IPV. Questions regarding marital relationships focused on marital norms and practices before and after displacement including related to polygamy, early and forced marriage, dowry as well as decision-making norms among couples, conflict within marital relationships and if and how marital conflict was addressed as well as physical and sexual IPV. The questions were written in English, translated to Somali and back translated to English by a different translator. They were piloted prior to data collection.

Three female and seven male Somali data collectors fluent in Somali and local dialects with experience either working for an NGO or conducting research were recruited from within Bokolmayo camp. Data collectors were supervised by a research supervisor hired from within the refugee community and a project team member (ST) directly supervised all data collection procedures and training. All data collectors completed a six-day training focused on protection of human subjects, qualitative methods and interviewing techniques***.*** They worked in pairs for IDIs and FGDs with one asking questions and the other taking notes. Interviews and FGDs were conducted by interviewers and facilitators of the same sex as the participants. All interviews were conducted in private settings and audio recorded. Interviews were between 40 and 90 min, while FGDs were between 120 and 220 min. A list of local medical, legal and other relevant support services was given to participants upon completion and referrals for psychological support were also provided.

### Ethical considerations

Verbal informed consent was obtained from all participants. Verbal, rather than written consent, was decided based on anticipated literacy and to minimize risks of a signed document revealing study participation. For participants aged 15–18, parental consent was not required as many families may have been separated during displacement and many women may have already been married between the ages of 15–18. Furthermore, seeking parental consent could have placed participants at risk if the nature of the study was disclosed. This study was reviewed and approved by institutional review boards at BIDMC in Boston, Massachusetts, as well as the Addis Ababa University in Ethiopia. Permissions to conduct the research were also obtained from UNHCR and ARRA. The onsite research supervisor, interviewers and facilitators were required to sign a code of conduct to ensure protection of study participants including confidentiality, integrity of data collection and fidelity to the study protocol. In the rare event that a data collector personally knew a participant, a replacement interviewer was assigned to the participant.

### Data analysis

Interviews and FGDs were transcribed verbatim in the original dialect and translated to English. These transcripts were uploaded into Dedoose (Version 7.0.23, Los Angeles, California), a qualitative analysis software system. A codebook was developed (VS, JS) a priori for the main analysis and used to code the transcripts. For the current analysis, a theoretical framework was developed based on literature review and identification of key factors related to marital practices among displaced populations, and additional codes were incorporated. The transcripts were reviewed and coded for themes (VS, AA, JS). This thematic analysis identified four prominent themes and numerous subthemes to capture the interplay of contextual, economic, social, and legal factors that have been changed by displacement and also influence marital practices in this population. Data were compared within different study groups to assess patterns related to specific themes. In addition, data from FGDs were compared with IDIs within each study group to identify or rule out dissenting perspectives.

## Results

In total, data were analyzed from 30 IDIs and 10 FGDs (*n* = 110 participants).

### Participant characteristics

Among participants, 30 (13 women, 17 men) were interviewed individually and 80 (31 women and 49 men) participated in FGDs (See Table 2). In the IDIs, participants ranged from ages 17 to 70 years, and the mean age of women was lower than men (29.1 years versus 36.8 years in men). The average length of time in the camp was 7.3 years (6.8 years for women, and 7.8 years for men). Most respondents were married, and all identified as Muslim. The average number of years of formal education across the entire sample was 5.6 years (4.3 years in women, and 6.6 years in men), but these data were skewed by education levels of the health workers and NGO staff who were interviewed. Similar characteristics were observed in the FGD participants (See Table 3).

**Table 2:** Demographic Data for In-depth Interviews

Participant Demographics	Refugee Community Members	Elders / Religious Leaders	Health Workers	UN / Non-Governmental Organizations	Community- Based Organizations	Policy-makers	Host Community Members	Total
	**N (%)**	**N (%)**	**N (%)**	**N (%)**	**N (%)**	**N (%)**	**N (%)**	**N (%)**
**# of Interviews**	16	4	2	2	2	2	2	30
**Nationality**								
Somali	16 (100)	4 (100)	2	0 (0)	2 (100)	0 (0)	0 (0)	24 (80
Ethiopian	0 (0)	0 (0)	(100) 0 (0)	2 (100)	0 (0)	2 (100)	2 (100)	6 (20)
**Sex**								
Female	8 (50)	0 (0)	0 (0)	2 (100)	1 (50)	1 (50)	1 (50)	13 (43)
Male	8 (50)	4 (100)	2 (100)	0 (0)	1 (50)	1 (50)	1 (50)	17 (57)
**Age** (mean, range, in years)	31.7 (17–62)	61.3 (51–70)	45.5 (45–46)	22.5 (20–25)	36.5 (32–41)	31 (19–43)	40.5 (37–44)	36.8 (17–70)
**Marital Status**								
Single	5 (31)	0 (0)	0 (0)	2 (100)	0 (0)	0 (0)	0 (0)	7 (23)
Married	11 (69)	4 (100)	2 (100)	0 (0)	2 (100)	1 (50)	2 (100)	22 (73)
Separated	0 (0)	0 (0)	0 (0)	0 (0)	0 (0)	1 (50)	0 (0)	1 (3)
**Length of Time in Camp** (mean, range, in years)	6.8 (0.3–9)	7.6 (7–8)	7.5 (7–8)	1.7 (1–2.5)	8 (8)	8 (8)	N/A	7.3 (0.3–9)
**Years of Education** (mean, range, in years)	3.6 (0–15)	2 (0–8)	15 (14–16)	15 (15)	10 (8–12)	7.5 (7–8)	2.5 (0–5)	5.6 (0–16)

**Table 3:** Demographic Data for Focus Group Discussions

FGD #	Participant Type	Total # of Women	Total # of Men	Country of Origin	Age Range (years)	Length of Time in Camp (years)
1	Male (15–25 years)	0	8	Somalia	17–25	1–8
2	Male & Female (26–45 years)	4	4	Somalia	27–45	6
3	Male (> 45 years)	0	8	Somalia	48–82	6
4	Female (15–25 years)	8	0	Somalia	17–25	7–8
5	Female (26–45 years)	8	0	Somalia	25–45	6–8
6	Female (> 45 years)	8	0	Somalia	45–49	5–8
**Other Groups**						
7	Clan / Religious Leaders / Elders	0	8	Somalia	25–80	6–8
8	Health Workers	1	7	Ethiopia	25–29	1–5
9	UN / Non-Governmental Organizations	2	6	Somalia & Ethiopia	21–49	1–6
10	Community-based Organizations	4	4	Somalia	27–50	8
	**Total**	**31**	**49**			

The following themes emerged from the analysis of IDI and FGD data: 1) Marital practices in Somalia prior to displacement, 2) Perceived changes in marital practices due to displacement, 3) Displacement-related factors that influence marital practices, and 4) Inequitable gender norms, power dynamics and IPV within marital relationships.

#### Marital practices in Somalia prior to displacement

Participants described several types of marital practices that took place in Somalia prior to displacement, summarized as:

1. Arranged marriage where the woman/girl is not consulted (forced by her parents)
2. Arranged marriage with consultation of the woman/girl (organized by her parents but with consent of both spouses)
3. Elopement where the woman/girl and man/boy like each other and run away together
4. Forced marriage initiated by a man/boy who rapes a woman/girl he wants to marry
5. Forced marriage initiated by a man/boy who together with his friends beats a woman/girl until she agrees to marry him
6. Practices that may overlap with any of the above: Polygamous unions (where a man marries more than one woman/girl), child marriage (where a girl is married before the age of 18), and dowry or bride wealth (transfer of wealth from the groom’s family to the bride’s family)

For example, (IDI 16, Male, 24 years) states: “*some girls escape with men they love, some others are asked by other family and they are given away, and some other girls are forced to marry without their will and are given to a person they don’t know*.” When asked why girls are forced he responded: “*They are forced because in our tradition when a girl reaches puberty age, she must be engaged. A Somali proverb says ‘girls must be married or buried’ and that’s our tradition.*” Several participants described forced marriages initiated by individuals external to the immediate family. For example, during a FGD with religious leaders, one participant described how a man, together with his male relatives or friends would carry out what is called “Dul Garaac”. First they would follow her, then: “*They engulf the girl and beat [her] with sticks until she goes out with them.*” (FGD 7, Male Religious Leader).

Child marriage was reported to be very common prior to displacement with most participants stating that 15 years of age is the appropriate time for a girl to get married. This age was commonly referred to as the age of maturity in Islamic religion; however, there were differences in what age was reported to be acceptable depending on whether the participant originated from a rural or urban region of Somalia. A number of participants reported the acceptability of girls getting married at a young age in Somalia. For example: “*The religion has said … that the lowest age [should] be at 9 years.* “(FGD 10, CBOs, 27–50 years). Most reported that the boys’ age of marriage should be higher, at least 20 years of age. Some explained that the later age at marriage for boys was related to the pursuit of education, which was not something considered important for girls. Several participants described that child and forced marriage in Somalia resulted from poverty.

Dowry or bride wealth, wherein wealth is transferred from the groom’s family to the bride’s family in order to confer a union and serve as a contract, was reported to be a common tradition in Somalia: “… *there is a Somali tradition that mostly commands to pay something when you want to marry a woman, that is an honorable norm to gratify her and her family … It is recommended to you to pay something to please her family and I think that this is a universal tradition…. previously it was usual to pay camel or livestock.*” (IDI 6, Male, 29 years). The tradition of dowry, called Yarad locally, was reported to be based on customary law with differing amounts based on the agreement between the two families. One male participant stated an estimate of a typical dowry: “*Five female camels, and if it is money, around one thousand US dollars, or 20 Million if it is converted to Somali Shillings. That is what is to be paid [to the bride’s family]*.” (IDI 12, Male, 25 years).

Polygamous unions were described as a frequent marital practice in Somalia. This type of marital practice co-existed with other practices described including child and forced marriage. Polygamy was reported to be supported by religion; a man could have up to four wives, but only if certain requirements such as equal treatment between the wives were met: *“Allah allowed men to have four [wives], but when they marry, they treat all the same.”* (IDI 3, Female, 18 years).

#### Perceived changes in marital practices due to displacement

Most participants described notable shifts in marital practices including decreased arranged and forced marriages, fewer child marriages, reduced dowry amounts and decreased polygamy in Bokolmayo refugee camp. There were few dissenting perspectives, with one religious leader and one 25-year-old man who felt that marital practices had not changed since displacement.

##### Arranged marriage

In the refugee camp, participants reported that couples more frequently choose their own spouses, a practice that was described as infrequent prior to displacement. Many participants noted that youth now get married without asking for the validation of their parents beforehand. There is more autonomy in deciding whom to marry and this is no longer primarily dictated or arranged by family members. For example, one participant stated: “*Her father used to force her without her consent... And now she comes with the man she loves, that is what changed.”* (IDI 10, Female, 19 years). A different participant commented: “*Earlier, mother and father were commanding the child and validating [them] to marry, now without notice of the father and mother they marry.”* (IDI 30, Female, 30 years).

These changes in marital norms were generally discussed in neutral or positive tones, especially among younger participants and those working with organizations in the community. Some of the older participants and religious leaders expressed resentment that parents were being ignored in this process and described it as disrespectful.

##### Forced marriage

A majority of participants indicated that forced marriage was less frequent in the camp compared to pre-displacement. On the other hand, several respondents stated that forced marriage was still occurring in the camp. For example, one female health worker explained the continued occurrence of forced marriage as being caused by economic needs: “… *Inside the camp, mostly the girl and boy choose each other. As it is now, also there is a marriage where the father is asked for the girl and engages her even if she is not pleased. Many cases of such kind happen, it passes us and they select the person from this camp, [because] now there are many necessities.*” (IDI 23, Female, 25 years, Health Worker).

In general, there seemed to be limited support of forced marriage. Several participants described forced marriage as an abuse or violation, while others, especially health workers and organizational staff, described negative consequences associated with forced marriage. For example, one participant stated:

“*Parents may call their daughter and may say that we have decided that your cousin [will] be your husband and [you will] marry him. The girl may not like that man (her cousin) and she may say, ‘father I do not want to marry this man’, and then her parents force her to obey them. Then they force her to marry and this marriage will be established without considering her future life and happiness within the marriage. This forcefully established marriage is a drawback of our culture. I saw many girls who were tortured by their husbands and their parents did not see it as a problem or said, ‘beat her and force her to obey you’. That is the problem of Somalis mostly.*” (IDI 22, Male, 45 years, Health Worker).

None of the participants described forced marriage by armed groups.

##### Child marriage

Participants typically described fewer child marriages, noting that the age of marriage is higher for girls in the camp compared to Somalia (i.e. 18 years of age compared to 15 years of age in Somalia). “*Girls that used to be married in childhood are married as adults now.*” (IDI 13, Female, 46 years). Incidents of child marriage were described as still occurring in the camp, as several organizational workers noted seeing cases, but there was general agreement that this marital practice was occurring less frequently than in Somalia. Several respondents were not supportive of delaying the age of marriage, while other participants agreed that delaying the age of marriage was advantageous for girls. Several noted the negative health consequences of early marriage including childbearing before the body is physically mature.

##### Dowry

It was widely agreed that the practice of dowry, though still considered an important cultural tradition, had changed due to economic hardship. While in Somalia the amount of the dowry may have totaled 1000 USD or multiple livestock, in the camps the amount was reported to be lower (18 USD) or even nothing if the individual is unable to afford it. “*Previously [we] used to pay camels or livestock but now it is not as usual. There are some changes. It depends on the financial ability of the man. It may be 500 Birr [18 USD] or whatever he can afford to gratify his in-law parents and the whole family of his beloved lady to build a good relationship with his in-law relatives.*” (IDI 6, Male, 29 years).

There were differing views on how shifts in dowry practices have impacted power and relationship dynamics within marriages. For example, one religious leader stated that transfer of dowry ensures good behavior on the part of the man. *“[Somalis] do believe that if the wealth is not taken from the man, he will not treat the woman well.”* (IDI 14, Male, 62 years, Religious Leader). Others expressed that the dowry confers an ownership of the woman and leads to poor treatment of the wife: *“…[in his] mind the wife in the house is like an animal and she belongs to him…[because] he paid a wealth.”* (IDI 15, Male, 45 years).

##### Polygamy

Polygamy was reported to have decreased since displacement. For example, one participant stated: “*Having many wives has reduced… Mostly men in the refugee camp don’t marry multiple wives*.” (IDI 14, Male, 51 years, Religious Leader). There were mixed perspectives on polygamy. Some participants stated that polygamy can cause breakdown of the family and instability, and is an injustice to the wives. Most stated that the main problems that arise from this type of family structure are economic in nature, or stem from not being able to treat the wives equally. The latter reportedly results in favoritism toward one wife and ultimately leads to jealousy and conflict.

Conversely, other respondents stated that polygamy is not problematic. For example, one religious leader stated that it is “*very good to marry many wives. We are Muslim people and it is a law to marry many wives.*” (IDI 14, Male Religious Leader, 51 years). On the other hand, one clan leader stated: “It was said that ‘a man who marries an additional woman has added a hell in his life’, it is a problem and to have one is most appropriate.” (IDI 20, Male Clan Leader, 66 years). While there were no clear attitudinal patterns by age or type of participant, some younger participants also expressed supportive views towards polygamy. For example, one 25-year-old man stated: “*the husband can have two, there are [men] who married one, some have three, Allah allowed men to have four, but when they marry, they treat all the same*.” (IDI 12, Male, 25 years).

#### Displacement-related factors that influence marital practices

Participants described a number of factors as being linked directly to perceived changes in marital practices as well as other issues that influence marital practices more generally. These factors include access to education, poverty, protection, security in and around the camp and the physical environment, NGO programming, laws on age of marriage and the social stigma of being unmarried.

##### Access to education

Almost all participants agreed that there is increased access to education for girls in the camp compared to in Somalia. “*In Somalia, girls were not educated, girls were not sent to school. They were discriminated against. But in the camp the school is open to both boys and girls.”* (IDI 17, Female, 30 years). Several participants also explained the link between marriage and education. For example, one responded noted: “*If a girl is married she halts her education.*” (IDI 21, Male, 45 years, Health Worker). A different respondent noted: “*Marriage has changed a lot…Now the girls are more likely in education [then getting married]*.” (IDI 13, Female, 46 years old).

Many respondents were very supportive of girls’ education, as illustrated by one participant’s statement: “*One educated woman is like educating the whole world.*” (IDI 24, Female, 20 Years, Organization Worker). Several participants described that attitudes had become more supportive: “*Earlier the community believed girls are for marriage, and there is no advantage for her to study. But recently this has changed and it is believed that girls do as the boys do. If she studies then later on she can work for her people and country.*” (IDI 19, Male, 18 years). However, a number of participants, including one male under 18 years of age, expressed that sending girls to school is a waste of time, and that it negatively impacts the girls’ mothers who then have more chores to do at home without any help. One respondent expressed fear in sending girls to school as they may be subject to harmful acts from boys, and have pregnancies that will bring shame on the family.

Access to schools in Somalia was reportedly limited because of few schools in rural areas, but also because of fear of armed groups. In one FGD with young women aged 15–25 years, one participant explained: *“We did not have confidence to learn because we were fearing for lives. For the sake of this fear we were not learning...We were fearing from Al Shabab network.”* (FGD 4, Female, 15–25 years). This contrasts with the conditions in the camp described by a different participant: *“once we had arrived here, everything is free, the education is free.”* (FGD 1, Male, 19–25 years).

##### Poverty

Poverty was universally stated as being a major concern of refugees in the camp, and was reported to have been exacerbated by displacement. The lack of employment and income generating activities in the camp make it hard for families to make ends meet. The increased economic hardship in the camp has reduced the size of the traditional dowry. Several respondents noted that forced and child marriages typically occurred because of poverty. However, almost no respondents cited financial gains for parents who marry girls at a young age in the camp, except under rare circumstances.

Polygamy was reported to have decreased due to the economic conditions in the camp. For example, one participant explained: “*Our law allows men to marry four wives, but this country where we live now makes restrictions to marry because of living conditions that restrict us to do that. And we are allowed to marry only one wife.*” (IDI 8, Male, 70 years, Clan Leader). Men are considered to have a duty to cover all household expenses: “*He has to bring money to the family; [he is] the one that buys clothes for his children and fulfills their needs. As a Muslim, [he] is expected to cover all costs*.” (FGD 8, Health Worker). This applies to polygamous families as well. But in the refugee camp, few men are able to afford more than one wife. *“I myself have now one wife and six children. We were together for 20 years and there is no other wife I married because my economic ability is not allowing me to do that.”* (IDI 22, Male, 45 years, Health Worker).

##### Security in and around the camp and the physical environment

Generally, participants described peace and safety in the camp compared to Somalia. One respondent stated: *“Our country has changed in different ways, it used to be violent, chaotic and unstable but here it is calm and stable.”* (IDI 12, Male, 25 years). A different respondent described the situation in Somalia: “*Very serious violence used to happen like robbing, looting… Thanks to Allah, we now have got peace [in the camp]*” (FDG 10, CBO). Despite general reports of peace in the camp, participants frequently noted that the physical environment in and around the camp contributes to higher risks of non-partner sexual violence toward women and girls compared to Somalia, especially when collecting firewood, and at water distribution points around the camp and in the host community. Schools were also noted to be a location where GBV occurs. Many participants noted that young girls aged 15–18 are particularly vulnerable to these risks. However, no participants specifically described early marriage as a strategy adopted to protect girls from the increased risk of sexual violence.

Despite these perceived risks, respondents described fewer restrictions for girls. For example one young woman stated: “*the girls go to work for their choice and go anywhere they want. That is empowering.*” (IDI 3, Female, 18 years). Respondents explained that there is more mixing of different clans and intermingling of populations originally from rural and urban areas in Somalia, and as well mixing of boys and girls at school. This may have contributed at least partially to the increase in love marriages as several respondents cited schools as places where new relationships may start. At the same time, a few participants described fear of unintended pregnancies in the camp as girls have more freedom and are going to school where they could interact with boys. At least two respondents described the need for girls to be married at the age of sexual maturity in order to avoid unintended pregnancies which could bring shame to families.

##### NGO awareness-raising programming

Many participants also discussed increased access to NGO programming in the camp, and how this programming has started to influence certain attitudes and behaviors. Some respondents attributed changes in marital practices, especially related to early and forced marriages, to awareness raising activities led by NGOs in the camp. For example, one respondent stated: “*In Somalia…the young girls used to marry by force without selecting the man...but when we have come here, there are organizations in the region, we are given awareness, they should not marry before 15 and once mature she should select the man she wants to marry.*” (IDI 17, Female, 30 years). A different participant expressed: “*There are many things that have changed. Like not marrying a girl who is under 18 years old. We received extra awareness from the different organisations in here. That is why I could say that many things have changed.”* (IDI 18, Male, 49 years). Another respondent stated: “*… in Somalia, girls at 15 years old were married…a lot of awareness programs were given to us and now it is rare to marry a girl less than 18 years old… sometimes you see teenagers getting married saying that it is their choice to get married at this age but now the community mostly approves of the awareness programs*.” (IDI 18, Male, 49 years).

At the same time, some male participants were at odds with these programs and explained that their own rights were being threatened.

##### Laws on age of marriage

A number of participants described the laws in Ethiopia around the minimum age at marriage as instilling fear among the refugees in the camp, especially among those who might have organized child marriages back in Somalia. For example, one respondent stated: “*Now they can’t be forced, fearing from Ethiopian law, and the refugees themselves [are] trained, many things are feared, the people who would force something are fearing*.” (IDI 21, Male, 46 years, Health worker). A different participant described why forced marriages no longer occur: “*We are in democratic country, there are NGOs and security, no one can force the girls to do things they don’t want, there is a government and rule of law here, girls are not forced to marry.”* (IDI 16, Male, 24 years).

Many participants made a distinction between religious or Islamic customary law which permits marriage starting at the age of 15 years and “democratic” or Ethiopian law which allows marriage starting at 18 years of age. Some respondents disapproved of this “democratic” law and described it as infringing on the role of parents. For example, one respondent explained how the new rules violate the parents’ traditional roles *“…you can’t manage your girl, you can’t let her [get] engaged before the girl is 15 or 18… all those consultations and counseling of the parents are violated, you yourself are violated.”* (FGD 7, Male Religious Leader).

##### Social stigma and marital status

Participants described the social value and importance that marriage confers women. As such, women described social stigma toward unmarried older women who were called “owls” by some respondents. Some interviewees described the social pressure to get married and stated that women who are not married by 20 or 21 years of age inherently must have a problem to not be well liked by men. On the other hand, some of these pressures may have become slightly relaxed in the camp, where respondents described more freedom in terms of when and how to get married. One respondent said that a woman in the camp could get married at age 40 if she so desires.

#### Inequitable gender norms, power dynamics and IPV within marital relationships

Most respondents described the persistence of inequitable gender norms since displacement and that the social position of women in Somali society is lower than that of men. The expected roles of men and women in marital relationships remain clearly demarcated, with overall agreement among women and men that a good wife in any type of marital relationship “*should be obedient and please her husband.*” (FGD 4, Female, 15–25 years). A number of respondents described marital discord and conflict as arising when a wife does not fulfill her duties and women were often noted to be the source of marital problems. For example, one participant expressed how women are the cause of conflict and violence: “*Problems arise from the side of women…[problems] always come because of wrong doing of women, that is how I believe.*” (IDI 6, Male, 29 years). Meanwhile, men were described as the key decision-makers who hold power within marital relationships. One participant explained why women aren’t involved in decision-making: “*Because a woman cannot make a decision but [only] men can. Women will become dizzy, how they can make a decision?”* (FGD 1, Male, 19–25 years). Another respondent stated: “*According to Somali culture, women have no power and there is no any decision they can make”.* “(IDI 6, Male, 29 years).

Participants described power differentials between men and women in relationships as a source of marital conflict. One male participant explained: “*Violence happens because they are women. Women and men don’t have the same power*” (IDI 29, Male from host community, 37 years). Such power differentials between men and women may be greater in child marriages where the girl is young, uneducated and unaware of her rights. For example, one respondent explained: “*In early marriages… the girl is immature, and misses her education and rights, surely there is violence.”* (FGD 10, CBOs, 27–50 years). There may be larger power imbalances within polygamous marriages as well: “*Most of the time men are interested in a second wife if he has one wife and he wants to get more children. Then he will marry a young girl*.” (IDI 16, Male, 24 years). However, no respondents suggested that the perceived declines in child and forced marriages and polygamy had changed marital conflict or violence. On the contrary IPV within marital relationships was reported to be commonly occurring in the camp, and numerous participants identified it as the most common form of violence facing women in this setting.

Physical and sexual IPV were noted to occur in all types of marriages, but certain marital practices were also linked with specific forms of violence. For example, polygamy was the marital practice most commonly linked to physical IPV. “*Men, according to Islam, are allowed to marry more than one wife… sometimes this is witnessed to be the cause of conflict*.”(IDI 8, Male, 70 years, Religious Leader). Polygamy was described as contributing to IPV in circumstances where the man does not treat his wives equally as mandated by the customary law. Favoring one wife over the other was described to lead to many problems including marital conflict which can eventually lead to physical IPV. Several respondents also described violence and conflict between wives in polygamous relationships and IPV perpetrated by wives towards their husbands in such relationships.

Forced marriage was also described by some respondents as being directly associated with physical IPV. For example, one respondent stated: “*A woman is forced [to marry] a man, then the man and the woman can not understand one another if they were not known to each other before their marriage. They don’t understand each other in the home. Then it may happen that the girl disappoints him in the house. And didn’t do as the man wants. And then he beats [her].*” (FGD 4, Female, 15–25 years). A different respondent explained: “*There are men who were forced to marry by their parents and also there are spouses chosen by themselves, the forced ones may treat each other badly*.” (IDI 11, Male, 17 years). A different respondent explained that there may be more violence in marriages which involved a dowry: “*Forced or arranged marriages cause conflicts, especially if there was money exchanged*” (IDI 22, Male, 45 years, Health Worker). Another respondent explained that dowry confers ownership of the woman and gives the husband license to perpetrate physical violence: ‘I beat her’, they are saying. ‘I paid for you too much money to your father… Then I have bought you.’ And then the girl is violated and beaten.” (FGD 6, Female, > 45 years).

Sexual IPV was described as commonly occurring within all types of marital relationships. It was not specifically described as being more frequent in early or forced marriages. Participants described incidents of forced sex within marital relationships which were linked to widely held norms around the role of a wife who is obligated to provide sex whenever desired by the husband. “*Her duties are … whenever he needs sexual intercourse she must be ready if it’s day or night*.” (FGD 2, mixed group, 26–45 years). These norms around sexual duties apply to all wives, regardless of age, type of marital union, or whether a dowry was exchanged. There was disagreement among participants about whether a woman could and should be allowed to refuse sex from her husband. “*Whether she refuses or not, it’s compulsory to have sex with her. But the organizations call it an abuse. And to me, she is my wife that [has to have] sex, I don’t see it as an abuse.*” (FGD 7, Male Religious Leader). A different respondent stated: “*She does not have a right to refuse if he has fulfilled his responsibilities and duties. She doesn’t have a right to refuse. It is the law*”. (IDI 19, Male, 18 years). A different participant expressed the belief that women have the right to say no to their husbands. If he forces her: “*It is like rape*” and the wife could file a complaint (IDI 24, Female, 24 years, Organizational Worker).

## Discussion

Findings from this qualitative study reveal reported shifts in marital practices, including perceived reductions in arranged and forced marriages, fewer child marriages, reduced dowry, and decreased polygamy among Somali refugees living in Bokolmayo camp in Dollo Ado, Ethiopia. Displacement has resulted in economic hardship for families and risk of non-partner sexual violence for girls and women remains high; however, there has also been a reported increase in access to education for girls and exposure to NGO awareness-raising activities, which along with Ethiopian laws prohibiting child marriage, have influenced decisions about marriage. Polygamy was also perceived to have decreased, primarily due to worsening economic conditions, and links between polygamous unions, forced marriages and dowry and physical IPV were described. While marital practices were perceived to have changed since displacement, the extent to which social norms related to these practices have shifted is unclear. There remain supportive attitudes towards child marriage and polygamy, including in some cases among youth, and attitudes that support gender inequitable norms and IPV persist in this population.

Our findings on child and forced marriage differ from other studies on child marriage in conflict or post-conflict settings [28–33] that suggest increased risk of child marriage after displacement. In northern Uganda, interruption of girls’ education, together with increased economic hardship, and lack of security contributed to decisions to marry at a young age to protect against non-partner sexual violence [28]. Furthermore, in that setting child marriage was also reported as a financial coping strategy for families after displacement, as wealth was conferred to the bride’s family as part of the dowry tradition [28]. Similarly, a separate study conducted in four settings (an IDP population in Northern Uganda, refugees from DRC in Uganda, Syrian refugees in Lebanon, and Somali refugees in Kobe refugee camp in Dollo Ado, Ethiopia) found that increased poverty after displacement influenced decisions to marry at a younger age, and that child marriage was believed to afford protection against sexual violence and unintended pregnancies [29].

While our study noted similar displacement-related conditions as previous studies, their influence on decisions about marriage differed. In our study, displacement was reported to be associated with increased poverty, but child marriage was not commonly described as a financial coping strategy for families. Instead, as a result of financial hardship, respondents reported that most men in the camp could not afford to pay a dowry. This suggests that there may be minimal economic benefit for families to support child marriage in this setting, and could explain the reported shifts in marital practices. However, a different qualitative study with 21 adult participants in Kobe camp in Dollo Ado (a camp neighboring Bokolmayo camp) reported that poverty was a driver of early marriage in this setting [29]. It is important to note that our sample was larger and more diverse than the sample in Kobe, and also included adolescents which may explain these differences. Importantly, in our setting, the displacement-related absence of dowry could also have other consequences, as it implies that marriages may be less formalized in this setting. A shift to informal marriages has previously been described as increasing vulnerability to emotional and physical IPV [28, 29].

Other factors that drive families towards child marriage in other contexts, such as risk of sexual violence, were also important concerns in Dollo Ado. For example, respondents described non-partner sexual violence as being common, but did not describe child marriage as a strategy to protect girls from sexual violence. This differs from other research including a study among Syrian refugees in Lebanon where parents reported higher risks of GBV in Lebanon than prior to displacement and described marriage as a means of protection against sexual harassment and violence and a way to preserve the honor of their daughters [32]. Another study conducted in Lebanon described heightened fears around protection of girls’ reputation and honor which depends on maintaining their virginity until marriage [31]. In our setting, it was not clear that the risk of sexual violence was perceived to have increased, and concerns about preserving the honor of daughters were not frequently described. Participants did report using alternative strategies to protect against the risks of sexual violence, in some cases this involved restricting movement of girls. However, it should be noted that there are geographical and cultural differences between refugees from Syria and Somalia which may explain the different findings related to sexual harassment and honor.

While in some humanitarian contexts displacement has reportedly interrupted girls’ access to education [28–33], in Bokolmayo refugee camp, girls’ access to education (at least at the primary-level) was reported to have increased compared to pre-displacement. This may be a protective factor against early marriage, as some participants reported that girls were now more likely to be in school rather than be married. Similarly, there were reported attitudinal changes towards education as well, with strong support for girls to complete schooling, though most respondents did not specify up to which level. Data from several other reports confirm increased access to primary education for Somali girls in Dollo Ado, Ethiopia [29, 47–49]. For example, UNHCR reported that primary school enrollment of refugee children doubled between 2012 and 2017, with over 47,000 refugee children in school at the end of 2017 [47]. The increased access to education reported in our study, together with positive attitudes about the benefits of education for girls may have contributed, along with other factors such as greater freedom of movement and autonomy about who to marry and when, to the reported increased age at marriage. The qualitative study in Kobe camp in Dollo Ado supports our finding that access to education for girls increased following displacement in this area, but suggests that this resulted in boys and girls mingling and influenced some parents’ decisions to undertake child marriage [29]. In our study, some participants described concerns related to increased interactions between boys and girls and of potential pregnancies but these were in the minority. Our findings are consistent with other research on the link between access to education and child marriage, including evidence demonstrating effectiveness of interventions providing incentives to continue education in delaying the age at marriage [50], as well as data suggesting messaging to shift social norms around early marriage among communities can be beneficial [51].

The legal environment in Ethiopia was reported to have played a role in the perceived reduction in incidence of child and forced marriages. Ethiopian law prohibits child marriage; the Revised Family Code of 2000 requires that both spouses provide full consent, and the Criminal Code of 2005 imposes a maximum prison sentence of three to 7 years depending on the age of the girl [51]. Most interviewees were aware of these laws. Many cited exposure to NGO activities raising awareness on minimum age of marriage laws as helping to influence decisions on child marriage and forced marriage. While some research suggests that laws requiring a minimum age of marriage may be associated with significantly lower prevalence of child marriage in sub-Saharan Africa [39], a number of other studies have reported limited evidence of the impact of such laws in the region [52, 53]. For example, one study of 10 African countries found inconsistent enforcement of child marriage laws, and limited correlation between strength of legislation and child marriage rates [52]. Many countries with such laws still permit children under 18 to marry with parental consent or make exceptions for customary law [54]. Furthermore, laws regulating age at marriage may have negative effects including driving the practice underground, or punishing young people [55]. However, despite limited evidence on the effectiveness of child marriage laws, such laws are important as they set the groundwork for advocacy efforts, and create an enabling environment for interventions targeting the root causes of child marriage [52, 56].

In addition to perceived declines in early and forced marriage, our findings also suggest perceived reductions in polygamy related to displacement. In this context, the reported reductions in polygamy appear to be driven primarily through increased poverty which makes multiple wives unaffordable, rather than through attitudinal change. Several reports suggest that in other humanitarian contexts polygamous unions have increased as a result of poverty [57, 58]. In Yemen, polygamy was indicated as a strategy by men to increase their income from begging conducted by multiple wives [57], while in Turkey, poverty in the refugee community resulted in polygamous marriages between Syrian women refugees and men in the host community [58]. However, It may be difficult to compare study populations and there may be different influences and beliefs around polygamy in each setting. In Bokolmayo, attitudes supportive towards polygamy persisted, and it was generally agreed that polygamy should be practiced only if a man has the resources to provide for all wives equally as dictated by religion and customary law. The lack of attitudinal change towards polygamy is perhaps not surprising given that there were no reported awareness-raising campaigns or NGO programs addressing this particular issue. In the future, should economic conditions improve such as through economic or livelihood interventions, it is unclear as to whether the practice of polygamy may also change.

Inequitable gender attitudes toward women were expressed by both women and men, and perceived power imbalances between men and women in marital relationships were noted. These power differentials may be even more pronounced when there is a large spousal age gap or forced marriage. Other research suggests that the same inequitable gender attitudes and norms that drive child and forced marriage may also perpetuate violence and that large spousal age gaps, power differences and lack of female autonomy which often characterize such unions are also IPV risk factors [19, 20]. In our setting, marital practices, in particular polygamy, forced marriage and dowry were perceived to contribute to physical IPV, which is consistent with other research [18–21, 42, 43, 59–61]. However, there is no evidence to suggest that the perceived declines in child marriage or polygamy might have contributed to reduced IPV in the camp. Rather IPV was reported to be occurring at high frequency. Our findings also suggest that sexual IPV was commonly occurring in all types of marital relationships, regardless of the woman’s age or exchange of dowry. In our study population, norms around sexual relationships in marriage and the absence of women’s right to refuse sex appear to be strongly held and were noted to apply to all married women regardless of age. Recent research across 34 countries examining the link between child marriage and IPV found a weaker association between child marriage and sexual IPV compared to physical IPV, but noted significant heterogeneity in Africa [18]. Somalia and Ethiopia were not countries included in that study, and further research is needed to further understand this relationship in different contexts.

Finally, in other research, changes in marital practices have been linked to other factors that are not specific to conflict or displacement such as urbanization [62], and access to mass media [46], as well as mobile phones and social media [35]. These broader factors may be at play among this population as well, though were not identified in this research as the interviews focused on perceived displacement-related changes to marital practices.

The study has several strengths. First, it included men and women as well as a wide range of ages and respondent types (married and non-married individuals, adolescents, religious leaders, service providers). This increased the diversity of the sample and ensured the inclusion of a wide range of differing perspectives. Second, the study included both individual interviews and focus group discussions. While focus groups discussions tend to elicit majority views and group norms, the individual interviews facilitated capture of dissenting perspectives. Finally, the study considered a spectrum of marital practices in the study design and analysis, recognizing that child marriage, forced marriage, and polygamy often co-exist.

There were also several important limitations to the research. The qualitative methodology employed does not permit for assessment of causal relationships between displacement and the factors examined, and similar data were not collected from non-displaced populations in Somalia which could have enabled additional comparisons. The analysis therefore focused on the perceptions of change of the participants who reported on their experiences and/or observations in the camp compared to prior to displacement. Social desirability bias could have influenced responses, but interviewers were recruited from within the camp and were trained in strategies to build trust and rapport with respondents. Nevertheless, the study adds to the gaps in the literature on marital practices including age of marriage, and the factors that may influence marital practices among displaced populations.

Our findings have implications for future programming and policy as well as research. Our study suggests that education for girls may be a protective factor against early marriage in humanitarian and refugee contexts, even within the context of financial hardship and high perceived risk of sexual violence. Efforts to increase safe access to education should be a priority in these settings as this could help to delay the age at marriage and have additional far-reaching benefits including decreasing IPV. In Dollo Ado, schools were reported as a site where GBV occurs, and there remain barriers to secondary education. Efforts to address these safety risks and obstacles to access are needed to ensure safe access for both girls and boys. Both NGO programming related to early and forced marriage and presence of laws on the minimum age of marriage were reported to contribute to perceived changes in practices. Thus, further efforts to strengthen community understanding of marriage laws and their enforcement, as well as interventions to change social norms around child and forced marriage are needed and should be tailored to the specific context. As economic factors, notably worsening economic situations, were linked to changes in marital practices (reduced polygamy and dowry) in this setting, livelihoods and economic interventions should take into consideration how they might influence marital age and assess unintended consequences. Marital practices such as polygamy and forced marriage were perceived to be associated with increased risk of IPV. Intimate partner violence prevention programs, especially in contexts where these practices are prevalent, should take this into account and incorporate specialized content. Sexual IPV was commonly reported, and laws recognizing forced sex within marital relationships are needed to help reduce sexual IPV. Further research is needed to better understand the underlying factors which influence marital decisions among displaced populations and to develop and test interventions to effectively prevent and reduce IPV in this population, especially among married adolescents. While targeted research, programs and policy are needed, it is important to note that child marriage, forced marriage, and polygamy co-exist and share similar risk factors.

## Conclusions

In summary, this qualitative study reports perceived reductions in child and forced marriages as well as polygamous unions and dowry among Somali refugees in Bokolmayo refugee camp in Ethiopia. Within a context of poverty, and high perceived risk of sexual violence both within the camp and in the host community, these reported reductions appear to be driven by NGO awareness-raising programming, the legal environment in Ethiopia as well as increased access to education for girls. Future programming should prioritize safe access to education in humanitarian settings in order to help delay age at marriage. Economic factors were strongly linked to shifts in marital practices and livelihoods interventions should consider and measure their impact on child marriage. Links between forced and polygamous marriages and physical IPV were reported and should be taken into consideration in IPV prevention programming. Laws recognizing sexual IPV within marital relationships are needed to reduce sexual IPV.

## Data Availability

Survey materials are available by request to the corresponding author.

## Abbreviations

AAU: Addis Ababa University
ARRA: Administration for Refugee and Returnee Affairs
BIDMC: Beth Israel Deaconess Medical Center
FGD: Focus Group Discussion
GBV: Gender-based Violence
IDI: In Depth Interview
IDP: Internally Displaced Population
IPV: Intimate Partner Violence
NGO: Non-governmental Organization
PLA: Participatory Learning Activity
UN: United Nations
UNHCR: United Nations High Commissioner for Refugees
WAHA: Women and Health Alliance International
